# Angiotensin-converting enzyme inhibitor reduces scar formation by inhibiting both canonical and noncanonical TGF-β1 pathways

**DOI:** 10.1038/s41598-018-21600-w

**Published:** 2018-02-20

**Authors:** Qing-Qing Fang, Xiao-Feng Wang, Wan-Yi Zhao, Shi-Li Ding, Bang-Hui Shi, Ying Xia, Hu Yang, Li-Hong Wu, Cai-Yun Li, Wei-Qiang Tan

**Affiliations:** 1 Department of Plastic Surgery, The First Affiliated Hospital, College of Medicine, Zhejiang University, Hangzhou, Zhejiang Province PR China; 2 Department of Plastic Surgery, The Fourth Affiliated Hospital, College of Medicine, Zhejiang University, Yiwu, Zhejiang Province PR China; 3 Department of Plastic Surgery, Sir Run Run Shaw Hospital, College of Medicine, Zhejiang University, Hangzhou, Zhejiang Province PR China; 4 Department of Hand Surgery, The First Affiliated Hospital, College of Medicine, Zhejiang University, Hangzhou, Zhejiang Province PR China

## Abstract

Angiotensin-converting enzyme inhibitors (ACEIs) can improve the fibrotic processes in many internal organs. Recent studies have shown a relationship between ACEI with cutaneous scar formation, although it has not been confirmed, and the underlying mechanism is unclear. In this study, we cultured mouse NIH 3T3 fibroblasts with different concentrations of ACEI. We measured cell proliferation with a Cell Counting Kit-8 and collagen expression with a Sirius Red Collagen Detection Kit. Flow cytometry and western blotting were used to detect transforming growth factor β1 (TGF-β1) signaling. We also confirmed the potential antifibrotic activity of ACEI in a rat scar model. ACEI reduced fibroblast proliferation, suppressed collagen and TGF-β1 expression, and downregulated the phosphorylation of SMAD2/3 and TAK1, both *in vitro* and *in vivo*. A microscopic examination showed that rat scars treated with ramipril or losartan were not only narrower than in the controls, but also displayed enhanced re-epithelialization and neovascularization, and the formation of organized granulation tissue. These data indicate that ACEI inhibits scar formation by suppressing both TGF-β1/SMAD2/3 and TGF-β1/TAK1 pathways, and may have clinical utility in the future.

## Introduction

Scar formation after injury is an unavoidable outcome of wound healing in adult mammal, and is characterized by persistent changes in the normal structure and function of the skin^1^. The overproliferation of fibroblasts and the overproduction of nonfunctional extracellular matrix components have deleterious consequences^1–3^. Skin scars, which range from barely visible fine white lines to disfiguring hypertrophic scars or keloids, are well documented^4–7^. A scar can cause cosmetic problems, but scarring can also result in the loss of joint function or hinder growth in children^8,9^. Psychological distress, including anxiety and depression, also often occurs^6,8,10,11^. Therefore, both patients and physicians should welcome even small improvements in scar management.

The lessons learnt from the treatment of fibrosis in internal organs have markedly advanced our understanding of scar management. It has been reported that angiotensin-converting enzyme inhibitors (ACEIs) improve the fibrotic processes in the heart, lung, liver, and kidney^12–15^. Two case studies also reported that low-dose enalapril (ACEI) improved postsurgical abdominal keloid scarring^16^. Hakan Uzun *et al*.^17^ reported that the early application of enalapril after dermal injury reduced scar formation in a rabbit ear wounding model. These results suggest that ACEI is an ideal treatment for scar management, but the underlying mechanism is not well understood.

The transforming growth factor-β (TGF-β) superfamily regulates many cellular functions, including cell growth, differentiation, adhesion, migration, and apoptosis^18^. Abnormal TGF-β signaling has been detected in increasing numbers of fibrotic and inflammatory conditions, including liver cirrhosis, renal fibrosis, systemic sclerosis, and hypertrophic scars^2,19–22^. TGF-β1 occurs almost ubiquitously in mammalian tissues, and the development of tissue fibrosis is primarily attributed to this protein^23^. There are two major pathways through which TGF-β1 propagates its signals. The canonical pathway involves the downstream effectors SMAD family member 2 and SMAD family member 3 (SMAD2/3), whereas the noncanonical pathway is closely associated with TGF-β-activated kinase 1 (TAK1)^24,25^. Because TGF-β1 signaling is so important in scar formation, we hypothesized that ACEI reduces scar formation by modulating TGF-β1-related pathways. Therefore, we investigated the underlying mechanism *in vitro* and *in vivo*.

Fibroblasts are the ultimate effector cells in scar formation^26^. Heemskerk *et al*. confirmed the presence of angiotensin-converting enzyme (ACE) in a NIH 3T3 fibroblast model^27^. Therefore, we first used mouse NIH 3T3 fibroblasts cultured with different concentrations of ACEI to evaluate the levels of fibrosis induced and the role of TGF-β1 signaling. We also confirmed our findings in a rat model of full-thickness skin wounds. Our results showed that the appropriate dose of ACEI induced the extracellular matrix to suppress the TGF-β1/SMAD2/3 and TGF-β1/TAK1 pathways, leading to the inhibition of scar formation. These findings suggest that ACEI can be used clinically to prevent scar formation.

## Results

### ACEI reduced fibroblast proliferation and collagen expression

**Figure 1:**
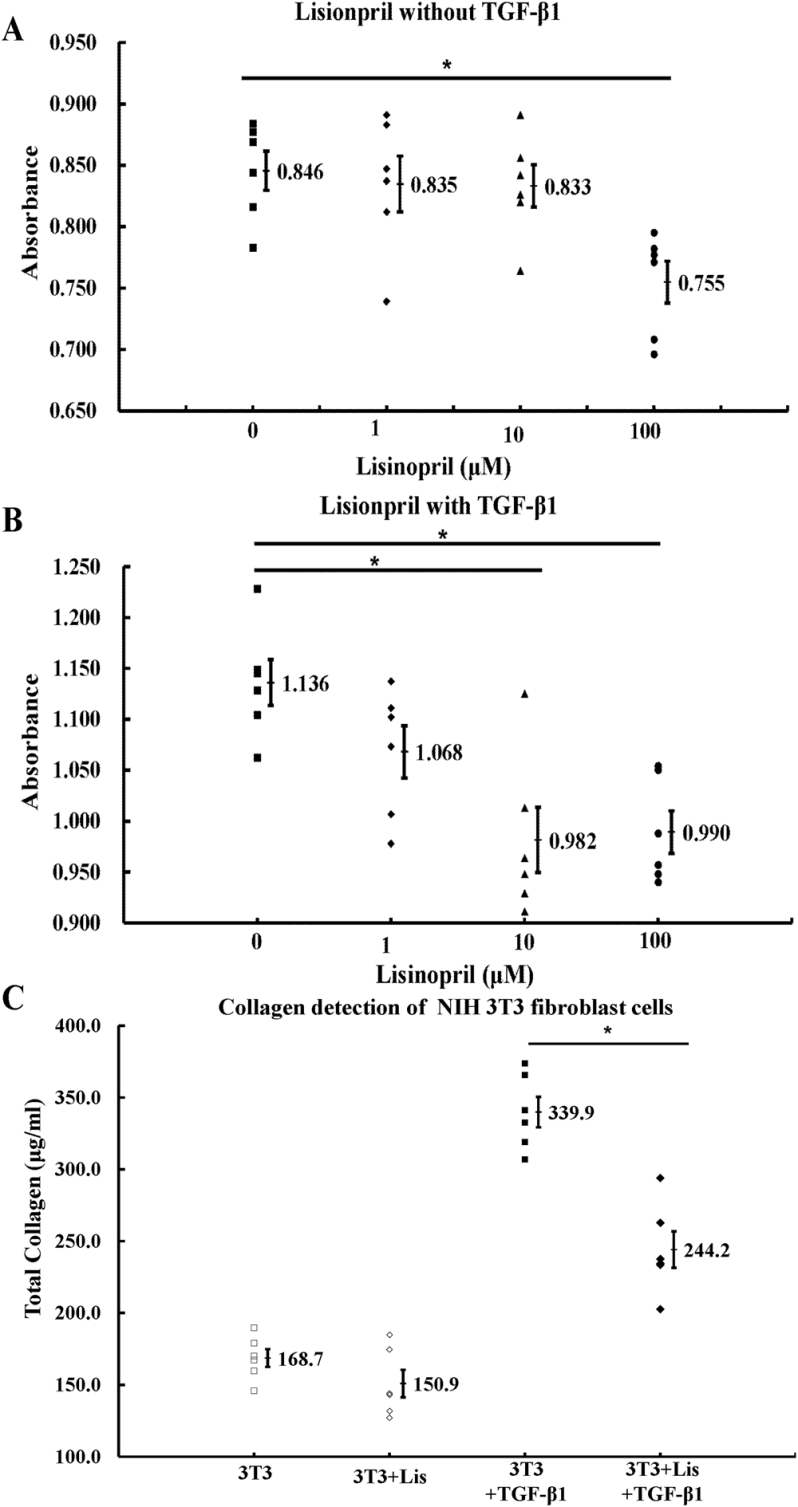
ACEI reduced fibroblast proliferation and collagen expression. (**A**,**B**) Proliferation of NIH 3T3 cells treated without (**A**) or with (**B**) 1.0 ng/ml TGF-β1 were measured with CCK-8. (**C**) Total collagen was detected in NIH 3T3 cells (3T3, NIH 3T3 cells; Lis, 10 μM lisinopril; TGF-β1, 1.0 ng/mL TGF-β1). NIH 3T3 mouse cells were cultured for 72 h in DMEM with 1% FBS. *P < 0.05.

To determine whether ACEI plays a significant role in cutaneous scar formation, we first studied mouse NIH 3T3 fibroblasts. The cells were cultured with various concentrations (0, 1, 10, 100 μM) of lisinopril for 24 hours. Cell proliferation measured with a Cell Counting Kit-8 (CCK-8). Lisinopril at a concentration of 100 μM inhibited cell proliferation more strongly than concentrations of 0, 1, or 10 μM, whereas the effect of 0, 1, and 10 μM lisinopril did not differ significantly (Fig. 1A). To better simulate physiological wounding, several groups of cells were also treated with 1.0 ng/mL TGF-β1 to simulate the traumatic stimulus. Interestingly, with the addition of TGF-β1, cell proliferation was also inhibited significantly by 10 μM lisinopril (Fig. 1B).

The total collagen in NIH 3T3 cells were detected with a Sirius Red Collagen Detection Kit. After culture for 72 h, the secretion of total collagen from the cells without lisinopril was higher than that from cells treated with lisinopril, with the stimulation of TGF-β1. However, the significant differences could not be found between the two groups without the stimulation of TGF-β1 (Fig. 1C).

### ACEI suppressed phosphorylation of SMAD2/3 and TAK1 *in vitro*

**Figure 2:**
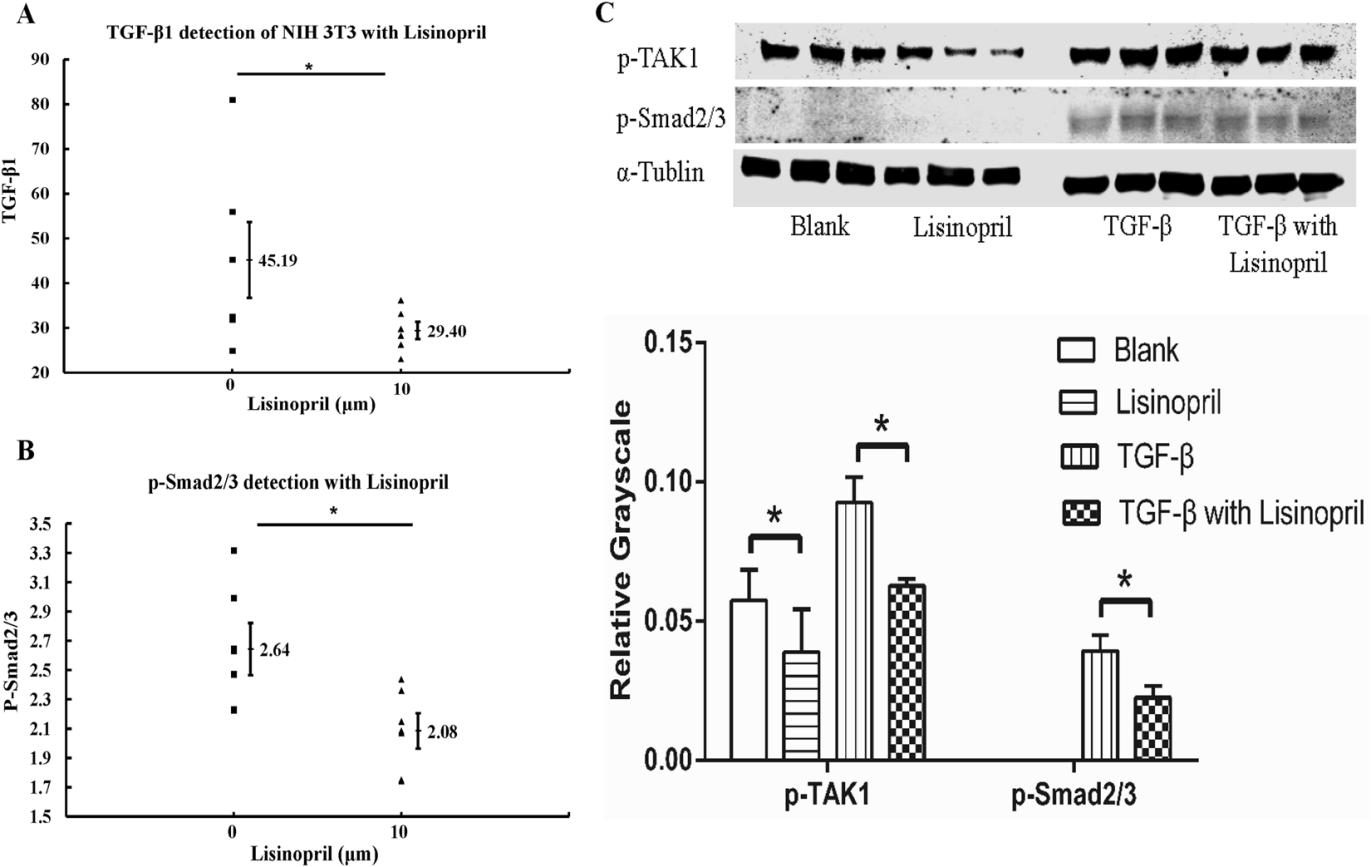
ACEI suppressed phosphorylation of SMAD2/3 and TAK1 *in vitro*. (**A**) TGF-β1 was detected in NIH 3T3 cells treated without or with 10 μM lisinopril. NIH 3T3 mouse cells were cultured for 24 h in DMEM with 1% FBS, then in Brefeldin A (a protein trafficking inhibitor; Selleck, Houston, Texas, USA) for 6 hours before the FACS. (**B**) Detection of p-SMAD2/3 in NIH 3T3 cells without or with 10 μM lisinopril. NIH 3T3 mouse cells were cultured for 24 h in the DMEM with 1%FBS, and then with 2 ng/mL TGF-β1 for 30 minutes before FACS analysis. (**C**) Western Blotting analysis of p-SMAD2/3 and p-TAK1 (Lisinopril, 10 μM; TGF-β1, 2 ng/mL). No p-SMAD2/3 was detected without TGF-β1 stimulation for 30 min before protein extraction. Full-length gel is shown in Supplementary Fig. 3. *P < 0.05.

To further clarify the mechanism underlying the ACEI-related downregulation of cell proliferation and collagen secretion, we used fluorescence-activated cell sorting (FACS) to quantify TGF-β1 expression. TGF-β1 decreased significantly in NIH 3T3 cells in the presence of 10 μM lisinopril (Fig. 2A and Supplementary Fig. 1). Because the canonical pathway of TGF-β1 signaling is closely associated with SMAD2/3^28–30^, we analyzed the levels of phosphorylated SMAD2/3 (p-Smad2/3), the biologically active form of the proteins. As expected, FACS showed that p-SMAD2/3 decreased in the cells in the presence of 10 μM lisinopril (Fig. 2B and Supplementary Fig. 2).

Experiments were performed to determine the involvement of both the canonical and noncanonical TGF-β1 signaling pathways in this phenomenon. One group of cells was cultured in normal nutrient medium supplemented with 10 μM lisinopril and stimulated with 2 ng/mL TGF-β1 for 30 minutes before the cell proteins were extracted. One group was cultured in normal nutrient medium supplemented with 10 μM lisinopril, without stimulation, and another group was cultured in normal nutrient medium without lisinopril but with TGF-β1 stimulation. The last group was cultured in normal nutrient medium with nothing added. Consistent with the FACS results, a western blotting analysis showed that with TGF-β1 stimulation, less p-SMAD2/3 was produced in the NIH 3T3 cells treated with lisinopril than that in the blank control group, whereas no p-SMAD2/3 was detected without TGF-β1 stimulation. The quantity of p-TAK1 was also lower in the NIH 3T3 cells treated with lisinopril than in the blank control group, with or without TGF-β1 stimulation (Fig. 2C and Supplementary Fig. 3).

### ACEI inhibited fibrosis and scarring in rats with acute dermal wounds

**Figure 3:**
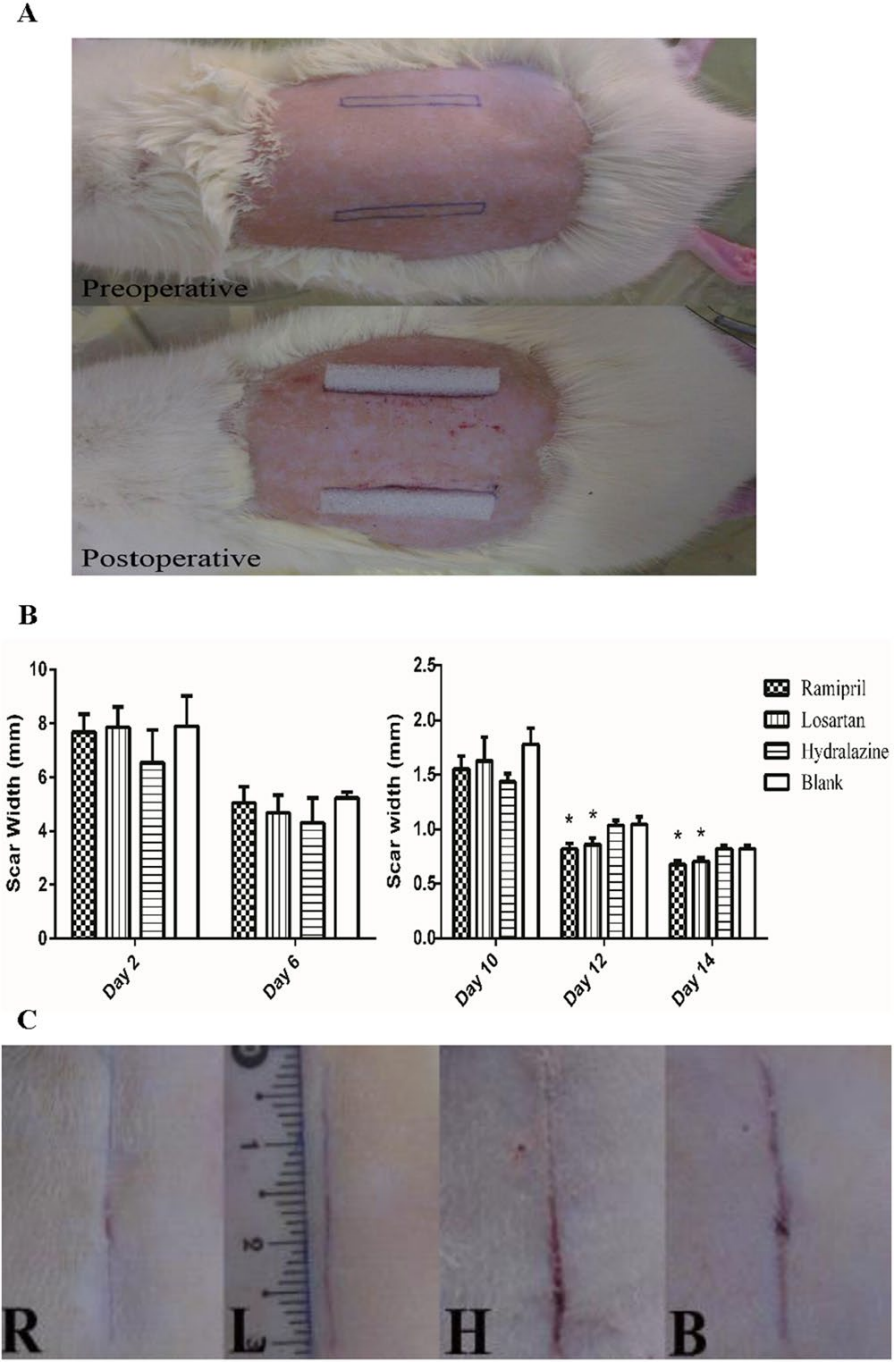
ACEI inhibited scar formation in acute dermal wounds in rats. (**A**) Representative photographs of the rat scar model. (**B**) Graphical summary of the changes in wound and scar widths (n = 12 wounds in six rats in each group). *P < 0.05 compared with the blank control group. (**C**) Representative photographs of the scar on day 14 after surgery. R: ramipril group; L: losartan group; H: hydralazine group; B: blank control group.

Based on the antifibrotic property of ACEI in NIH 3T3 cells, we next investigated the potential antifibrotic activity of ACEI in a rate model of acute dermal wounding. Two sections of skin were surgically removed from the backs of rats (Fig. 3A). Because the early application of a drug improved its performance^17^, the rats were randomly assigned to receive either ramipril (ACEI), losartan (angiotensin receptor blocker, ARB) or hydralazine (another type of blood-pressure-lowering agent) immediately after dermal injury. The control rats received only vehicle (water). We used lisinopril to treat the cultured cells because it is more soluble in water than other ACEIs, whereas we chose ramipril for the *in vivo* experiments because it has a relatively longer half-life than other ACEIs. The wound and scar widths were measured in all four groups throughout the healing process. The scar width did not differ significantly in all four groups on postoperative days 2, 6, or 10, but in postoperative days 12 and 14, the scar width was significantly narrower in the ramipril and losartan groups than in the other groups (Fig. 3B).

**Figure 4:**
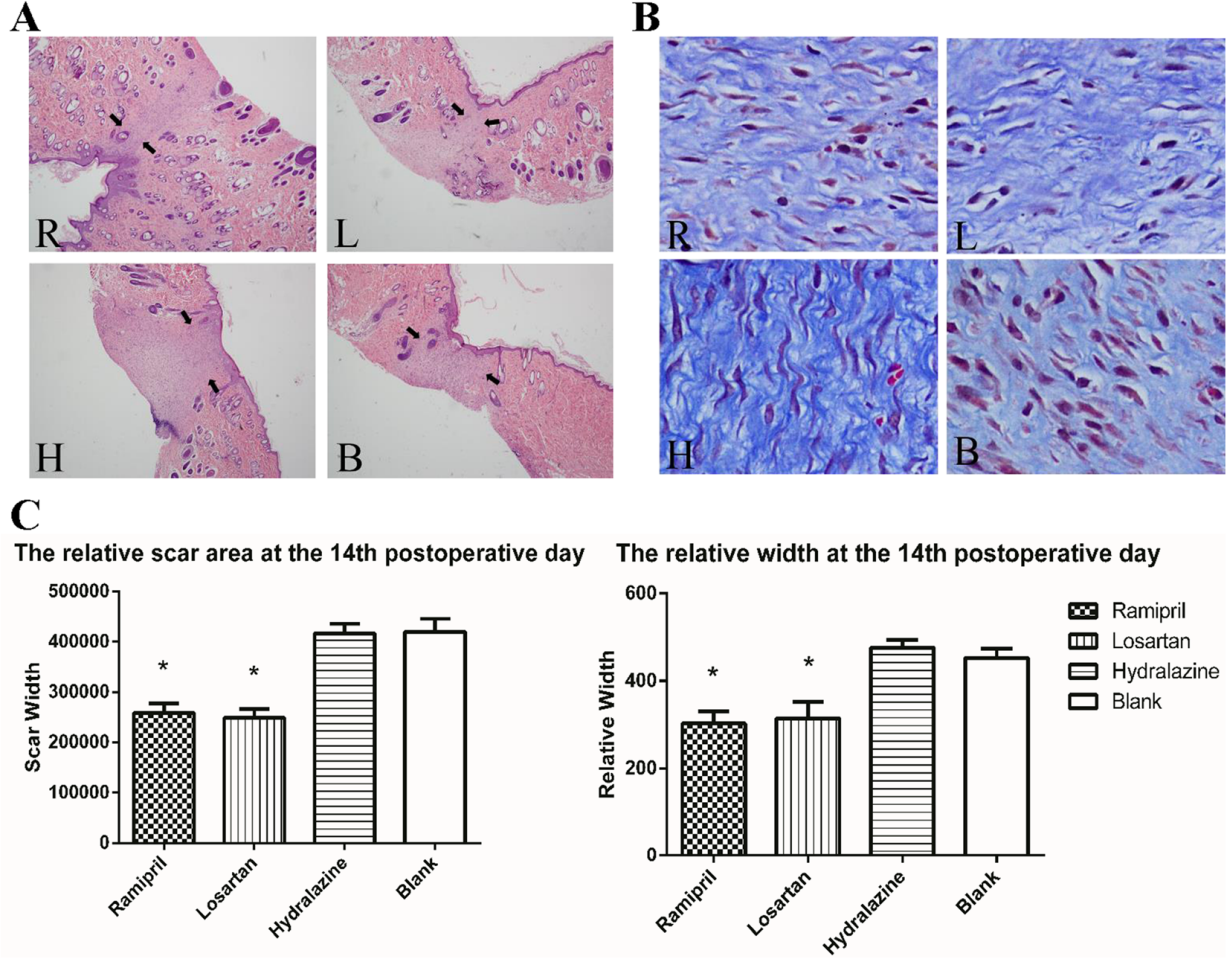
ACEI inhibited fibrosis and scarring in a rat scar model. (**A**,**B**) Representative photomicrographs of scar tissue obtained on day 14 with H&E staining (**A**) or Masson staining (**B**). Black arrows (**A**) mark the scales of scars. R: ramipril group; L: losartan group; H: hydralazine group; B: blank control group. (**C**) Relative scar areas and relative widths in the four groups (n = 12 wounds in six rats in each group). *P < 0.05 compared with the blank control group. H&E staining; images were obtained 40× magnification with an Olympus CKX41SF inverted phase-contrast microscope. Areas were calculated with the Image-Pro Plus v. 6.0 software (Olympus, Japan).

The wounds in all four groups were completely epithelialized within 14 days, after which the animals were killed and the scar tissues were collected (Fig. 3C). A microscopic examination on the final day revealed that the scars in the ramipril and losartan groups were not only narrower, but also showed better re-epithelialization and neovascularization than those in the other groups, and the formation of organized granulation tissue was apparent (Fig. 4A). Masson staining showed that the ramipril and losartan groups had loosely arranged collagen fibers and fewer fibroblasts, whereas the hydralazine and blank control groups had dense, irregular collagen fibers and more fibroblasts (Fig. 4B). Consistent with the gross measure of scar widths, the relative scar area and width determined with a histological examination were smaller in the groups treated with ramipril or losartan (Fig. 4C).

### ACEI inhibited SMAD2/3 and TAK1 pathways *in vivo*

**Figure 5:**
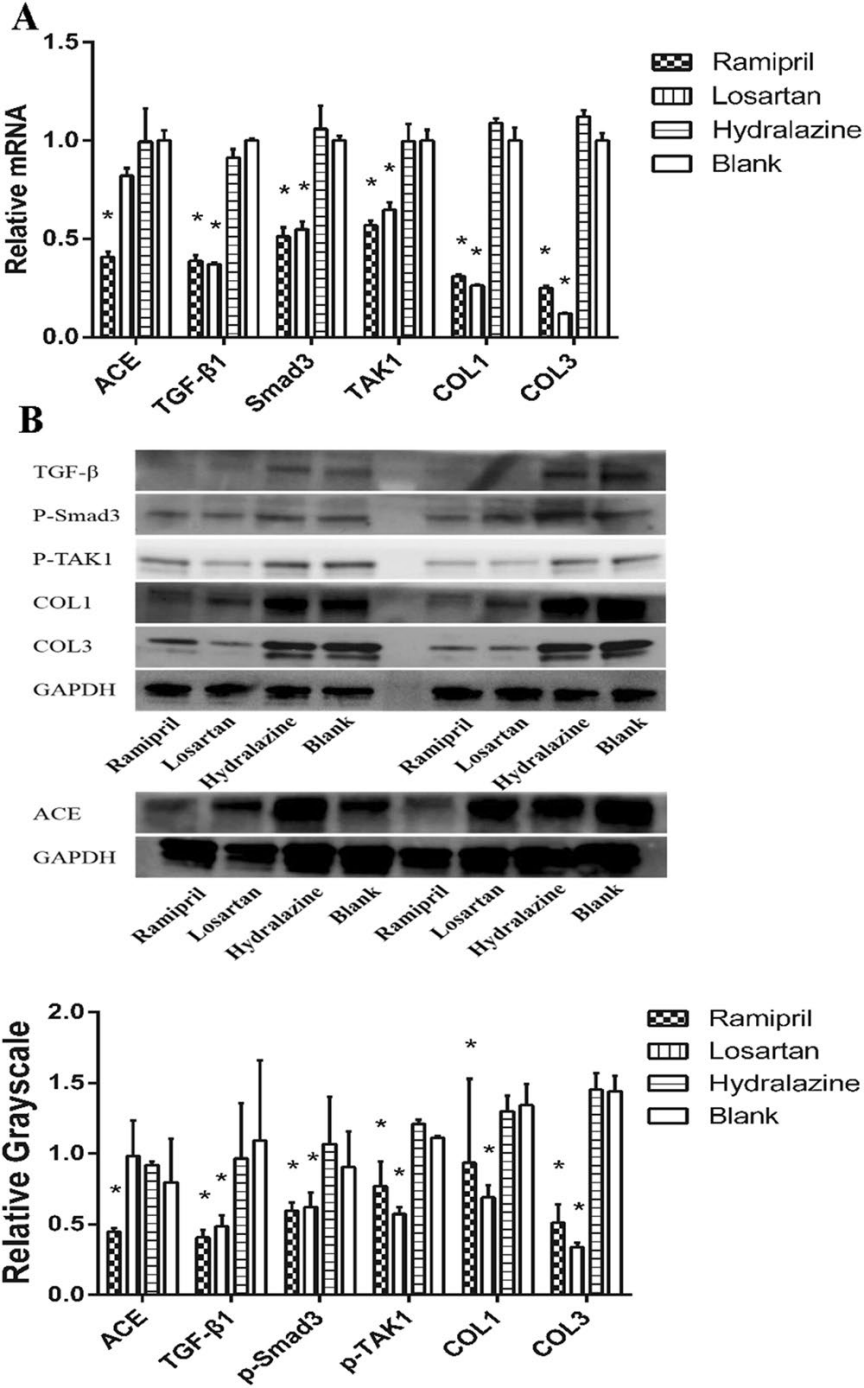
ACEI inhibited SMAD2/3 and TAK1 pathways *in vivo*. (**A**) Expression levels of ACE, TGF-β1, SMAD2/3, TAK1, collagen I and collagen III mRNA in scar tissues. (**B**) Western Blotting analysis of TGF-β1, p-SMAD2/3, p-TAK1, collagen I and III, and ACE in scar tissues. Full-length gel is shown in Supplementary Fig. 4. *P < 0.05 compared with the blank control group.

Having established the ACEI-associated downregulation of TGF-β1 signaling in murine fibroblasts, we then investigated the potential mechanism *in vivo*. Real-time reverse transcription (RT)-PCR showed that the levels of SMAD2/3 and TAK1 mRNAs were significantly lower in the scar tissue of the ramipril and losartan groups than in those of the hydralazine and blank control groups (Fig. 5A). A western blotting analysis also showed that the levels of p-SMAD2/3 and p-TAK1 proteins were lower in the groups with less scar formation (Fig. 5B and Supplementary Fig. 4). As expected, the mRNA and protein levels of TGF-β1, collagen I and collagen III were statistically lower in the tissues of the ramipril and losartan groups than in those of the other two groups.

## Discussion

Here, we have presented strong evidence of the effect of ACEI on wound healing and scar formation in rat dorsal wounds, and have demonstrated the crosstalk between ACEI and TGF-β1 both *in vitro* and *in vivo*. The improvement in scar formation was associated with reductions in fibroblast proliferation, collagen deposition and TGF-β1 expression and led to a more normal structure of the healing skin. The improved skin parameters were associated with the downregulated phosphorylation of SMAD2/3 and TAK1, which indicated the inhibition of both the canonical and noncanonical TGF-β1 pathways. Our results are consistent with previous reports on the effects of the RAS on myocardial infarction, which demonstrate the efficacy of ramipril in reducing fibrosis and collagen accumulation^31,32^. Our results also suggest that the beneficial effects of the RAS blockade induced by ACEI probably also extend to ARBs, such as losartan.

Most cells are involved in wound healing, including lymphocytes, neutrophils, macrophages, and fibroblasts. Fibroblasts are the major mesenchymal cell type in connective tissue, and are recruited to the injured site where they deposit collagen and elastic fibers^33^. They provides many regulatory mediators to the microenvironment and thereby contribute to the maintenance of wound healing and scar formation^34,35^. Our first study suggested that ACEI reduced the proliferation of murine NIH 3T3 fibroblasts, especially when the cells were stimulated with TGF-β1. During physiological wound repair, but not in tissue fibrosis, myofibroblasts are transiently present and are removed by the initiation of the apoptotic machinery^36^. Therefore, the proper inhibition of fibroblast proliferation can reduce abnormal wound healing and the development of fibrotic diseases^37^.

TGF-β1 signaling is significantly involved in many phases of wound healing, including inflammation and angiogenesis^38–40^. Our study has shown that ACEI inhibits the phosphorylation of SMAD2/3 and TAK1, which are two mediators of TGF-β1 signaling. The canonical signaling pathway for TGF-β1 involves the SMAD family^25^. Upon phosphorylation by TGF-β1 receptors, SMAD2 and SMAD3 form heteromeric complexes with co-SMAD or SMAD4. The SMAD2-SMAD3-SMAD4 complex is then translocated into the nucleus, where it regulates the transcription of TGF-β1 target genes^25,41,42^. Distinct from the activation of SMAD-dependent cascades, a predominantly SMAD-independent signaling pathway that mediates the profibrotic effects of TGF-β1 operates *via* TAK1^24^. TAK1 is involved in the TGF-β1–induced expression of type I collagen and fibronectin by activating the MAPK kinase(MKK)3/p38 and MKK4/JNK signaling cascades, respectively^24^. Our study demonstrated that ACEI inhibits both pathways of TGF-β1 signaling, reducing fibroblast proliferation and collagen deposition, leading to a more normal structure of the healing skin.

TGF-β1 is considered as the most important target in scar management because it supports excessive disorganized collagen deposition^43^, which is consistent with our findings in microscopic observation. Although reducing the expression of TGF-β1 by gene transfection or antibodies has been demonstrated experimentally^1,44^, no medicinal product is available for routine use. TGF-β1 regulates the expression of multiple genes related to fibrosis *via* both the canonical and noncanonical pathways. Therefore, the simultaneous inhibition of SMAD2/3 and TAK1 by ACEI is a promising strategy for blocking TGF-β1 signal transduction. No significant differences in bodyweight or physical condition were observed after the administration of different drugs to the rats in this study. These results indicate that ACEI significantly inhibits TGF-β1-induced scar formation *in vivo*, but does not significantly affect the general health of rats.

Our *in vivo* study also investigated the impact of ARBs, specifically losartan. Clinically, both ACEIs and ARBs have yielded similar results in terms of blood pressure control and cardiovascular protection^45,46^. ACEIs and ARBs differ pharmacologically in their mechanism of action and the levels at which they block the RAS. Although ARBs block RAS distally, at the level of the angiotensin II type-1 (AT1R), ACEIs block the conversion of angiotensin I to angiotensin II and thus reduce the amount of available angiotensin II to bind to either AT1R or angiotensin II type-2 receptor (AT2R)^47^. Both AT1R and AT2R are upregulated in human cutaneous wounds^48,49^, and AT2R is expressed more strongly than AT1R within the area of scarring^49^. Enhanced ACE expression is still detectable in cutaneous human scars 3 months after wounding^48^. However, the beneficial effects of RAS blockade are quite confusing. In AT1R-knockout mice, wound healing was delayed relative to that in the controls^50^, perhaps resulting from the disruption of the inflammatory phase and the impairment of the transition to proliferation and remodeling^51–53^. The knockout of AT2R accelerated healing but impaird quality^54^. The effect of AT2R on rate of wound closure may depend on the phase of wound healing in which it is applied. Recently, Abadir *et al*. suggested that the use of topical ARBs is an effective treatment for chronic wounds, whereas ACEI was not^47^. However, our study shows that both ACEI (ramipril) and ARB (losartan) positively affected wound healing and scar formation when orally administered. Our results are also supported by other recent studies that have shown that the oral treatment of diabetic rats or mice with ACEI or ARB accelerated wound healing^55,56^. The discrepancies among these studies might be explained by the differences in the metabolism of the drug and their tissue distributions, as well as by their systemic effects (e.g., on blood pressure, heart rate and the immune system).

In conclusion, our findings provide powerful insights into the inhibition of scar formation by ACEI. Orally administered ACEI normalized the structure of healing skin, resulting from the dual inhibition of SMAD2/3 and TAK1 signaling. The effects of ramipril on the deposition and arrangement of collagen in healing wounds may open a new avenue for the use of ACEI in fibrotic skin diseases.

## Methods

### Cells preparation

Mouse NIH 3T3 fibroblasts (American Type Culture Collection) were incubated at 37 °C under 5% CO_2_/95% air. The cells were cultured in Dulbecco’s Modified Eagle’s Medium (DMEM) supplemented with 10% (vol/vol) fetal bovine serum (FBS). The concentration of FBS was reduced to 1% at 24 h or 72 h before testing. Some groups of cells were cultured with ramipril (Sigma-Aldrich, St. Louis, MO, USA) and/or TGF-β1 (to simulate the traumatic stimulus). Cell proliferation was detected with the Cell Counting Kit-8 (CCK-8; Dojindo, Japan). Total collagen was detected by the Sirius Red Collagen Detection Kit (Chondrex Inc., Washington, USA). After reading the Manual carefully, we performed our test according to the instructions and procedures.

### Flow-cytometric (FACS) analysis

Allophycocyanin-conjugated anti-TGF-β1 antibody (TW7-16B4) was from BioLegend (San Diego, CA, USA). Alexa-Fluor®-647-conjugated anti-p-SMAD2/3 antibody (O72-670) was from BD Pharmingen (San Jose, CA, USA). To stain intracellular p-SMAD2/3, we used a rabbit polyclonal antibody directed against p-SMAD2/3 (Cell Signaling Technology, Danvers, MA, USA), followed by phycoethrythrin-conjugated goat anti-rabbit IgG antibody (SouthernBiotech, Birmingham, AL, USA), with fixation and permeabilization buffers (eBioscience, San Diego, CA, USA). The stained samples were analyzed on a Beckman Coulter CyAn ADP flow cytometer (Beckman Coulter, Fullerton, CA, USA), and the data were analyzed with the FlowJo software (Tree Star, Inc., Ashland, OR, USA).

### Rat preparation

All animal experiments were approved by the Zhejiang University Animal Care Committee. The experimental protocols and animal care were performed according to the guidelines for animal experiments of the Institutional Animal Care and Use Committee of Zhejiang University. Male Sprague Dawley rats (8–12 weeks old, average weigh 225 g) were used in the study (Laboratory Animal Center of Zhejiang University, Hangzhou, China). Randomization was performed by an independent central statistical unit. Ramipril (8 mg/kg bodyweight per day; Merck, Whitehouse Station, NJ, USA), losartan (50 mg/kg bodyweight per day; LKT laboratories, St. Paul, MN, USA), or hydralazine (40 mg/kg bodyweight per day, Sigma-Aldrich, St. Louis, MO, USA) was dissolved in water and administered orally to the rats at the same time every day.

### Rat animal model

The rat was anesthetized with 7% Chloral Hydrate (0.5 ml/100 g) using peritoneal injection. A thin rectangle of skin (3 × 0.3 cm^2^) parallel to but 1.5 cm away from the midline, was excised on both sides of dorsal skin, so each rat had two small portions of skin removed. The incision was made through the dermis and subcutaneous fascia, exposing the underlying muscle (which is not cut). A 3 cm (length) × 4 mm (width) × 5 mm (height) trimmed gelatin sponge was inserted into the excised wound. Wound care with 70% ethanol was performed on days 2 and 4 after surgery, and the widths of the wounds or scars were measured with a sliding caliper after surgery. Fourteen days after the operation, the rats were anesthetized with isoflurane inhalation, and the scars were harvested for analysis. The scar widths were grossly measured, and we also calculated the relative scar areas and relative widths with microscope observation. The tissue were stained with hematoxylin and eosin, and images were obtained at 40× magnification with an Olympus CKX41SF Inverted Phase Contrast Microscope. The areas were calculated with the Image-Pro Plus v. 6.0 software (Olympus, Japan).

### Western blotting analysis

Scar tissues or NIH 3T3 cells were homogenized in RIPA buffer (Thermo Scientific, Scotts Valley, CA, USA) mixed with a protease inhibitor cocktail (Thermo Scientific), loaded on a 4–12% gradient bis-Tris gel for electrophoresis (Invitrogen, Carlsbad, CA, USA), and transferred electrophorentically to a polyvinylidene difluoride (PVDF) membrane with a conventional method, as described previously^57^. The membrane was blocked (Odyssey, Pomona, CA, USA) and incubated with a rabibit anti-p-SMAD2 (Ser465/467)/SMAD3 (Ser423/425) antibody (D27F4; Cell Signaling Technology), a rabbit anti-p-TAK1 (Ser412) antibody (Cell Signaling Technology), and a mouse anti-α-tubulin antibody (Developmental Studies Hybridoma Bank, Iowa City, IA, USA) at 4 °C overnight, and then reacted with fluorescein-isothiocyanate-conjugated goat anti-rabbit or goat anti-mouse antibody (Li-Cor, Lincoln, NE, USA). The fluorescence intensities were visualized by Odyssey CLx Infrared Imaging System (Li-Cor). The fluorescent signals were captured and stored digitally, and quantified with Image Studio Lite ver. 4.0 (Li-Cor).

### Real-time RT-PCR

To analyze the mRNA levels in the scar tissues, total RNA was extracted using the RNeasy Mini Plus kits (Qiagen, Valencia, CA, USA). The purified RNA (200 ng) was reverse transcribed with a high-capacity iScript cDNA synthesis kit (Bio-Rad, Hercules, CA, USA). The expression levels of TGF-β1, SMAD2/3, TAK1, collagen I and collagen III, and glyceraldehyde 3-phosphate dehydrogenase (GAPDH) mRNAs (with primers from Applied Biosystems) were analyzed with quantitative real-time RT-PCR using an ABI OneStepPlus^TM^ Real-Time PCR System (Applied Biosystems, Carlsbad, CA, USA). GAPDH was used as the internal control.

### Histological analysis

Wound beds surrounded by a margin of unwounded skin were collected at day 14 after injury (n = 12 wounds in six rats in each group). The wounds were divided in half in the least-healed portion. They were then fixed overnight at 4 °C in 60% methanol, 30% chloroform and 10% acetic acid. The tissues were processed through a graded series of ethanol and embedded in paraffin blocks. Tissue sections of 5 μm were stained with stained with hematoxylin–eosin (H&E) or Masson’s trichrome to visualize neotissue formation, collagen deposition and the amount of neovasculature.

### Statistical analysis

The *in vitro* experiments were repeated with at least five batches of NIH 3T3 fibroblasts and the *in vivo* experiments were repeated three times, and qualitatively similar data were obtained in all repetitions. Data were analyzed with the SPSS, version 19.0 software (SPSS Inc., Chicago, IL, USA). Values are expressed as the mean ± SEM. Comparisons among groups were made with one-way analysis of variance (ANOVA), followed by a least-significant-difference (LSD) or Student-Newman-Keuls (SNK) test (more than 2 groups) or a 2-tailed unpaired Student’s t-test (two groups). A value of P < 0.05 was considered significant. As stated above, observers and statisticians were blinded to the group identity during all quantifications.

## Electronic supplementary material

supplementary figures
