# Supplementary material for: Angiotensin-converting enzyme inhibitor reduces scar formation by inhibiting both canonical and noncanonical TGF-β1 pathways
Source: Sci Rep. 2018 Feb 20;8:3332. doi: 10.1038/s41598-018-21600-w (PMC5820264; doi:10.1038/s41598-018-21600-w)

Angiotensin-converting enzyme inhibitor reduces scar formation by inhibiting both canonical and noncanonical TGF-β1 pathways

Qing-Qing Fang[1,2,3], Xiao-Feng Wang[1,2,3], Wan-Yi Zhao[1,2,3], Shi-Li Ding[4], Bang-Hui Shi[1,2,3], Ying Xia[1,2,3], Hu Yang[4], Li-Hong Wu[1], Cai-Yun Li[2] & Wei-Qiang Tan[1,2,3]*

[1]Department of Plastic Surgery, The Fourth Affiliated Hospital, College of Medicine, Zhejiang University, Yiwu, Zhejiang Province, PR China.

[2]Department of Plastic Surgery, The First Affiliated Hospital, College of Medicine, Zhejiang University, Hangzhou, Zhejiang Province, PR China.

[3]Department of Plastic Surgery, Sir Run Run Shaw Hospital, College of Medicine, Zhejiang University, Hangzhou, Zhejiang Province, PR China

[4]Department of Hand Surgery, The First Affiliated Hospital, College of Medicine, Zhejiang University, Hangzhou, Zhejiang Province, PR China.

Corresponding author at:

Wei-Qiang Tan, M.D. Department of Plastic Surgery, The Fourth Affiliated Hospital, College of Medicine, Zhejiang University, N1 Shangcheng Road, Yiwu, Zhejiang Province 322000, P.R. China.

Email address: tanweixxxx@zju.edu.cn

Supplementary figure 1

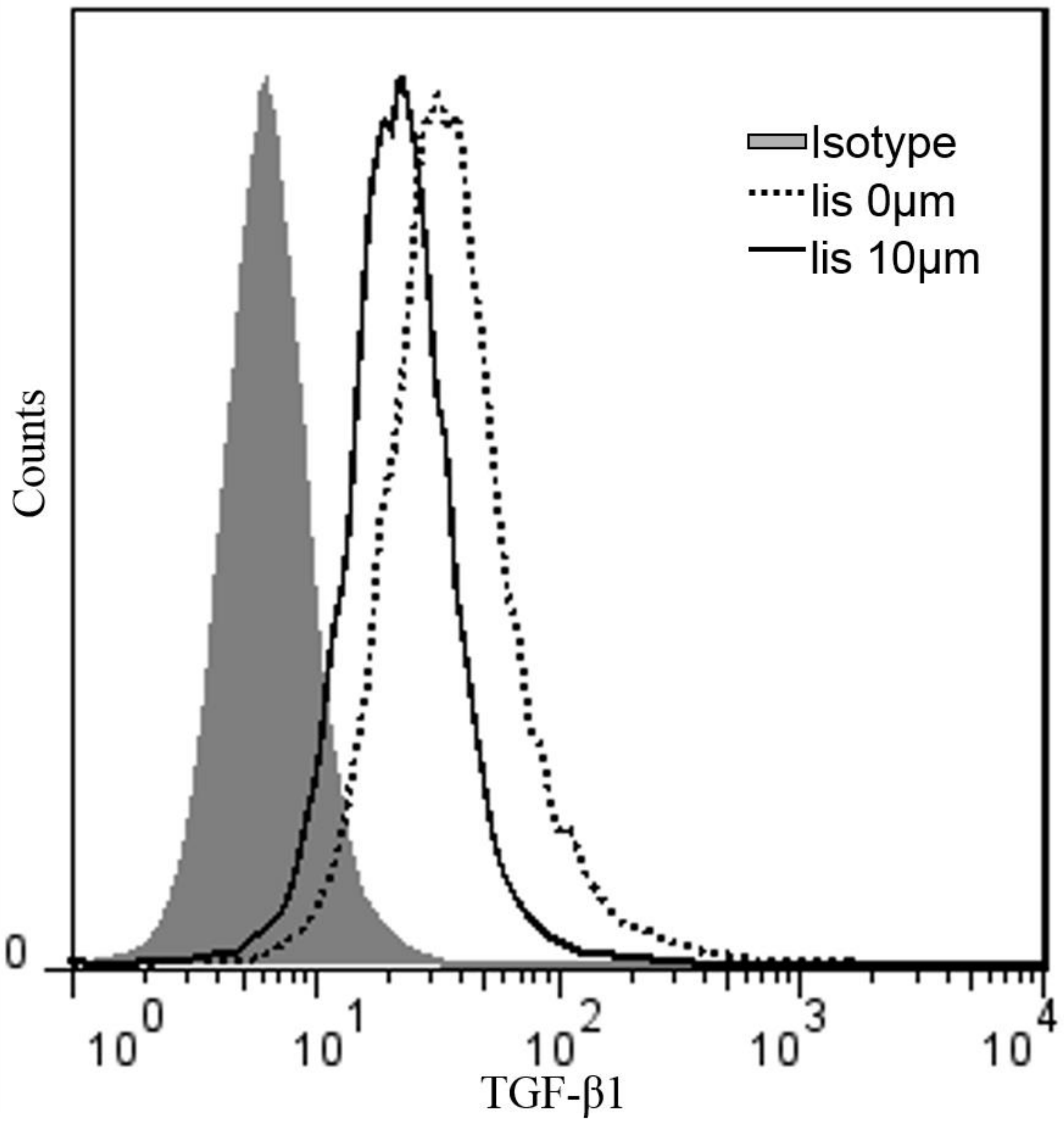

Supplementary Figure 1. TGF-β1 detection of NIH 3T3 cells without or with 10 μM Lisinopril. The NIH 3T3 mouse cells were cultured 24 hours in the DMEM with 1%FBS, then in Brefeldin A for 6 hours before the FACS.

Supplementary figure 2

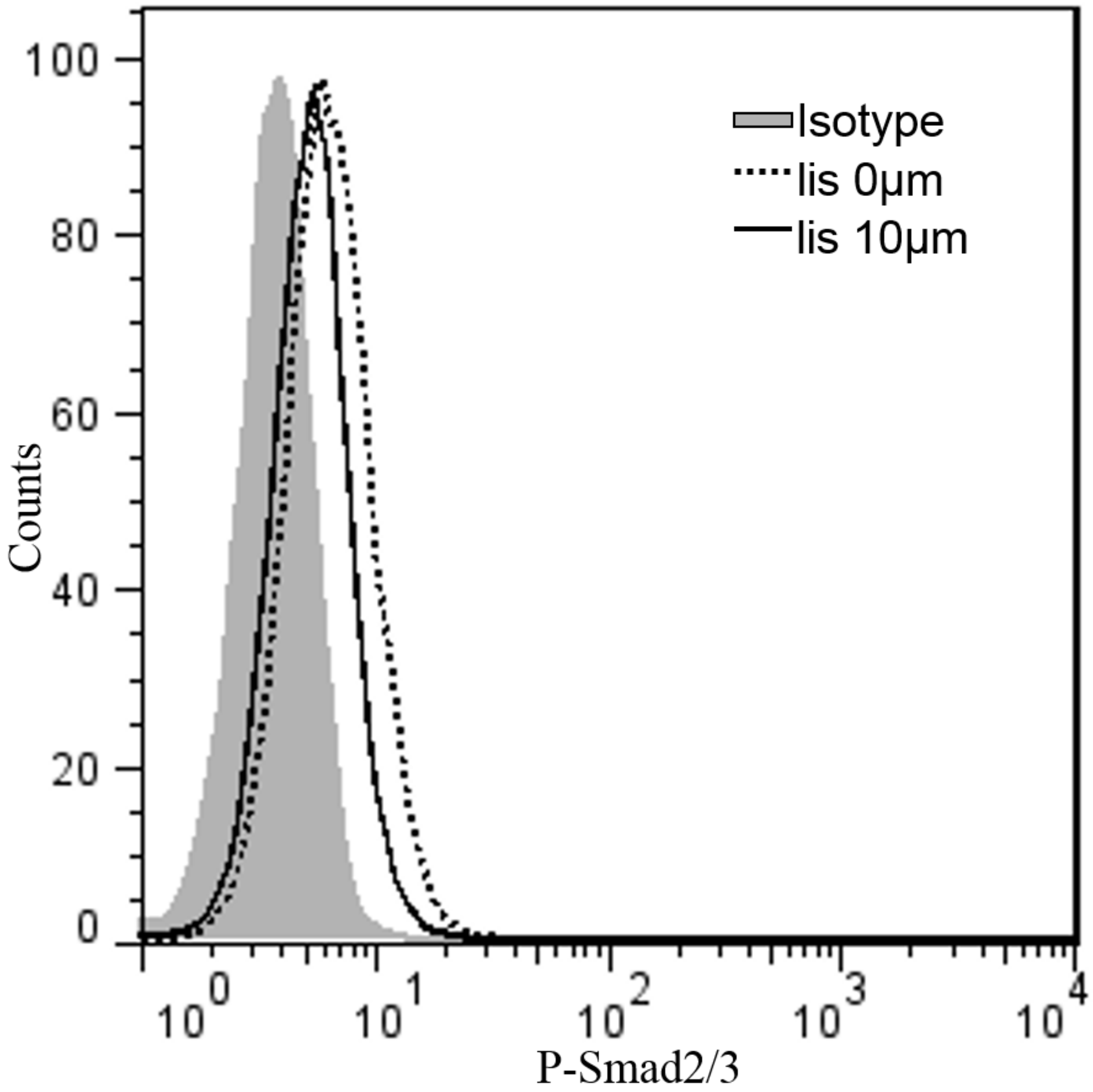

Supplementary Figure 2. P-Smad2/3 detection of NIH 3T3 cells without or with 10 μM Lisinopril. The NIH 3T3 mouse cells were cultured 24 hours in the DMEM with 1%FBS, then in 2 ng/ml TGF-β1 for 30 minutes before the FACS.

Supplementary figure 3

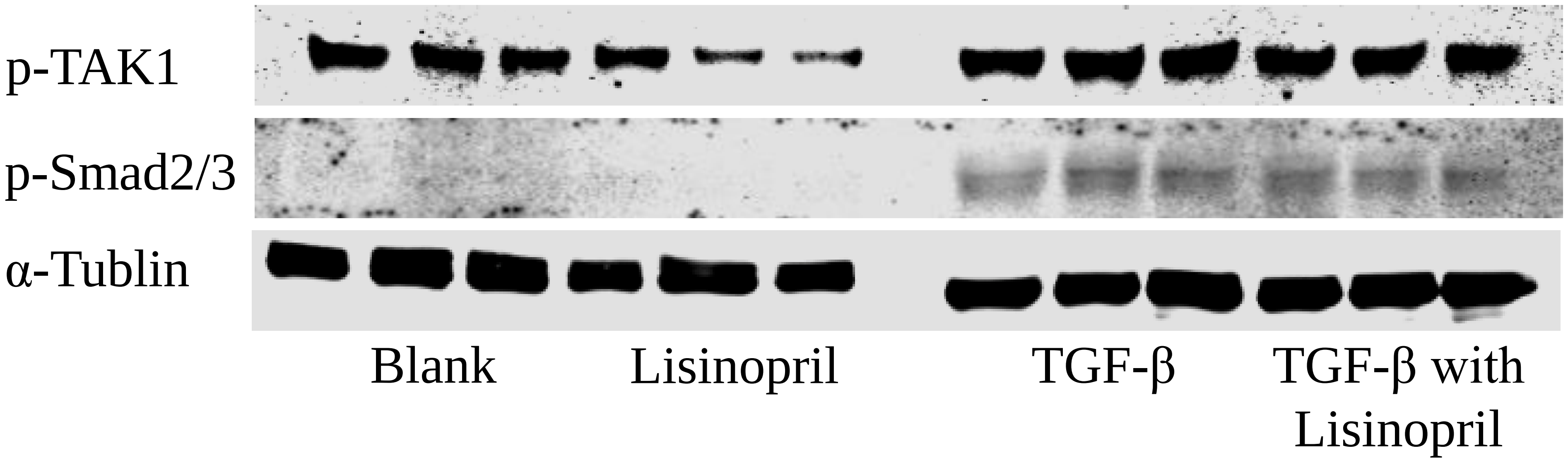
p-TAK1
p-Smad2/3
α-Tublin
Blank
Lisinopril
TGF-β
TGF-β with Lisinopril

p-TAK1

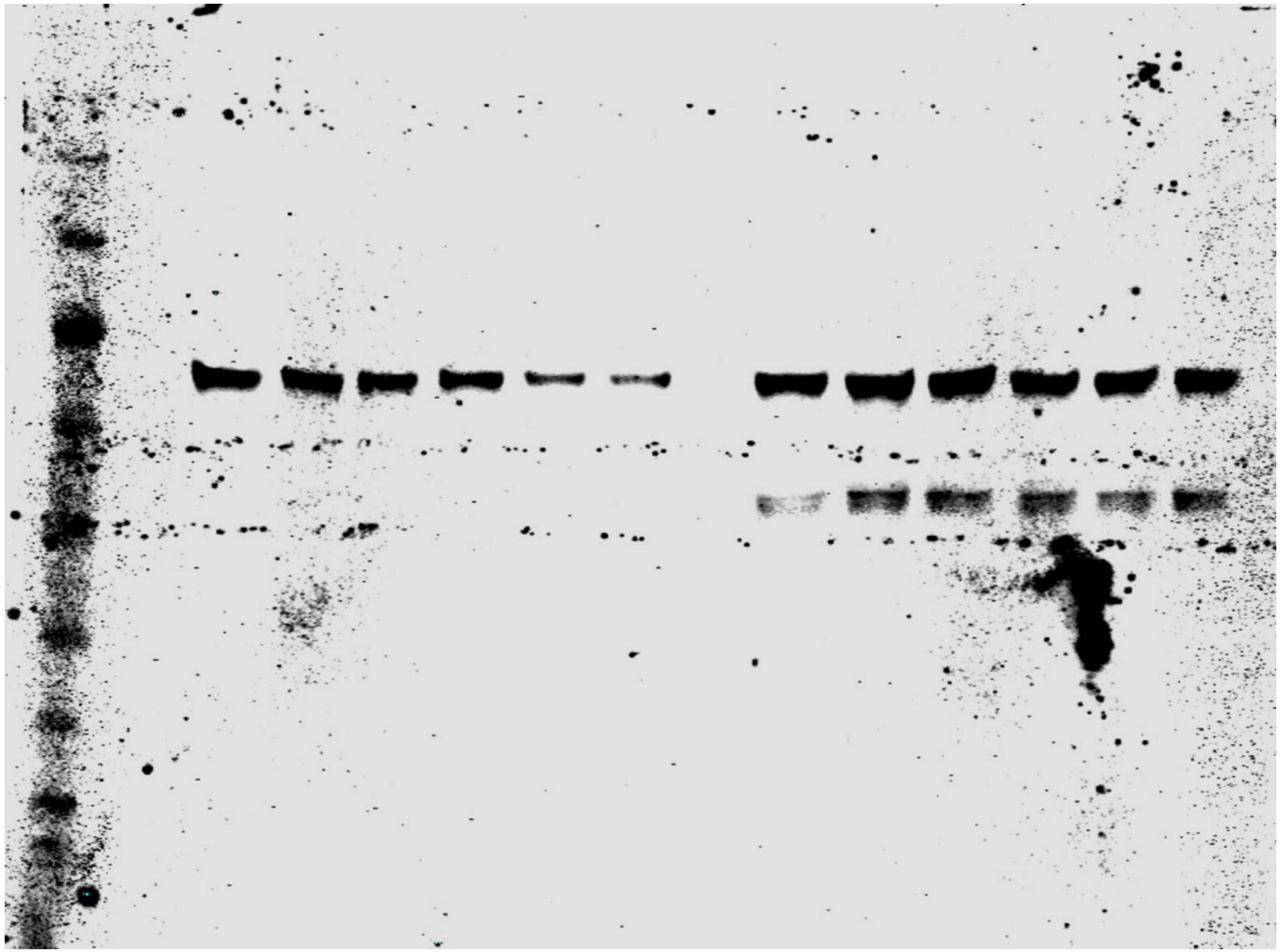

p-Smad2/3

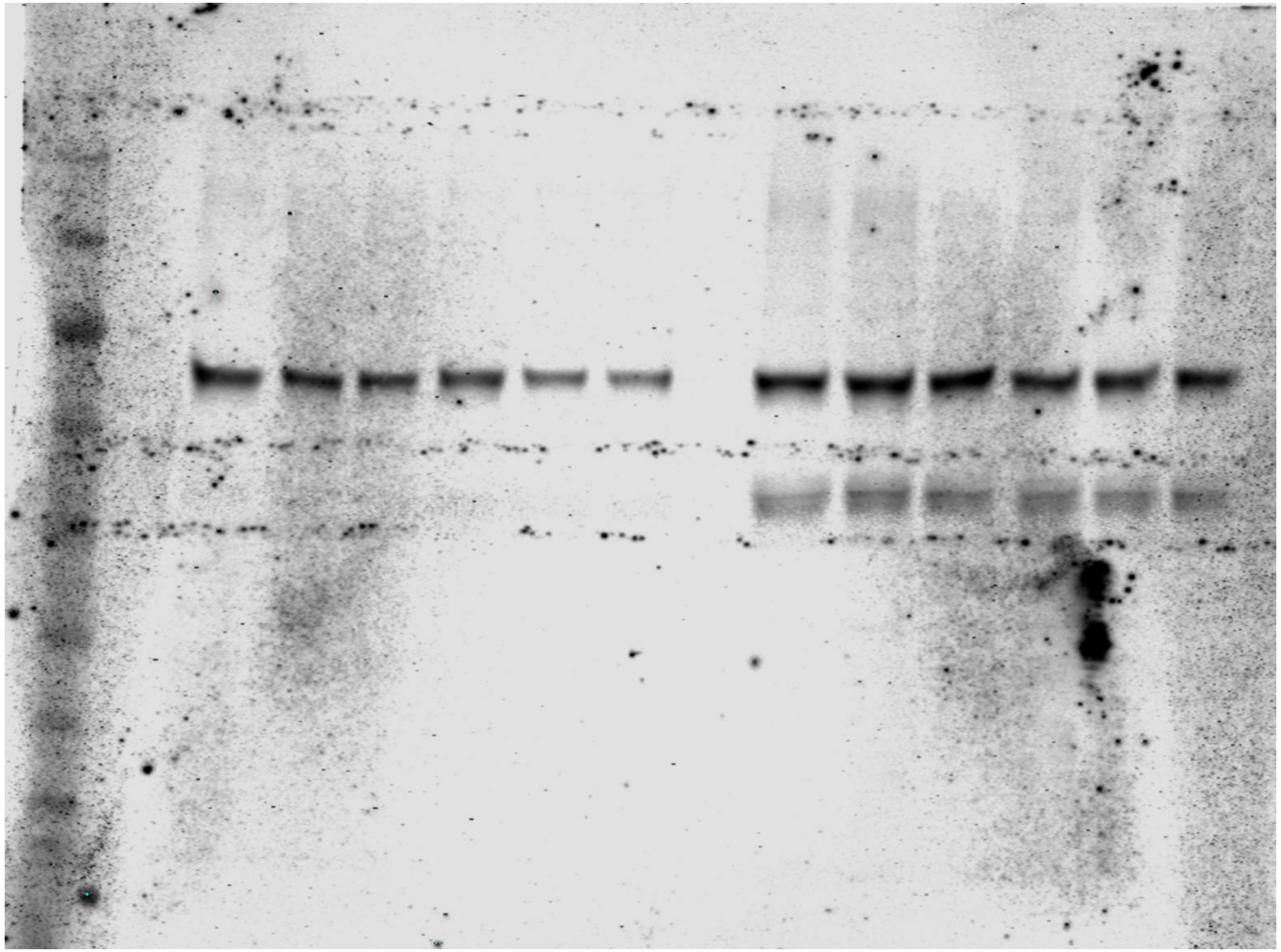

α-Tublin

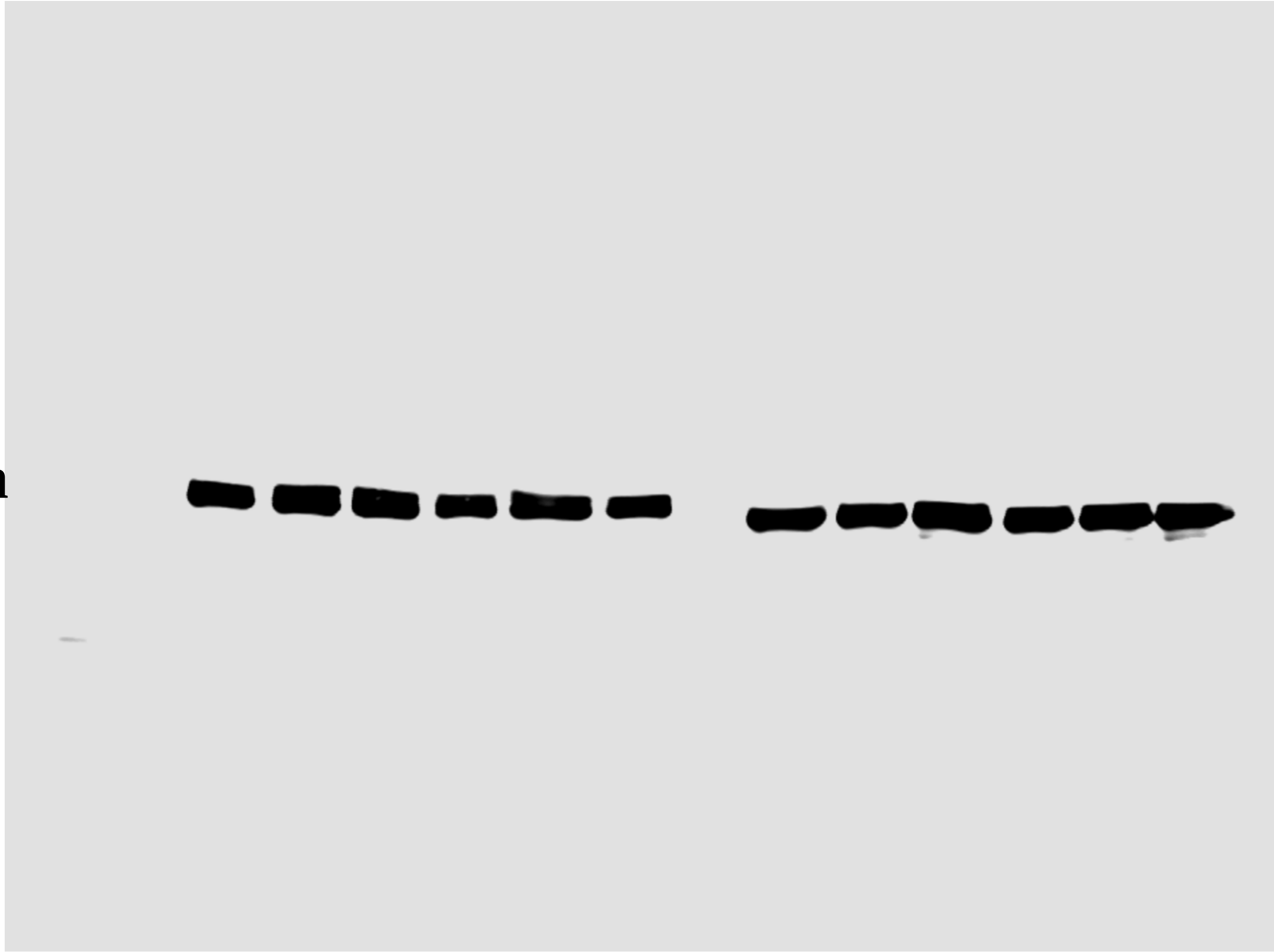

Supplementary figure 4

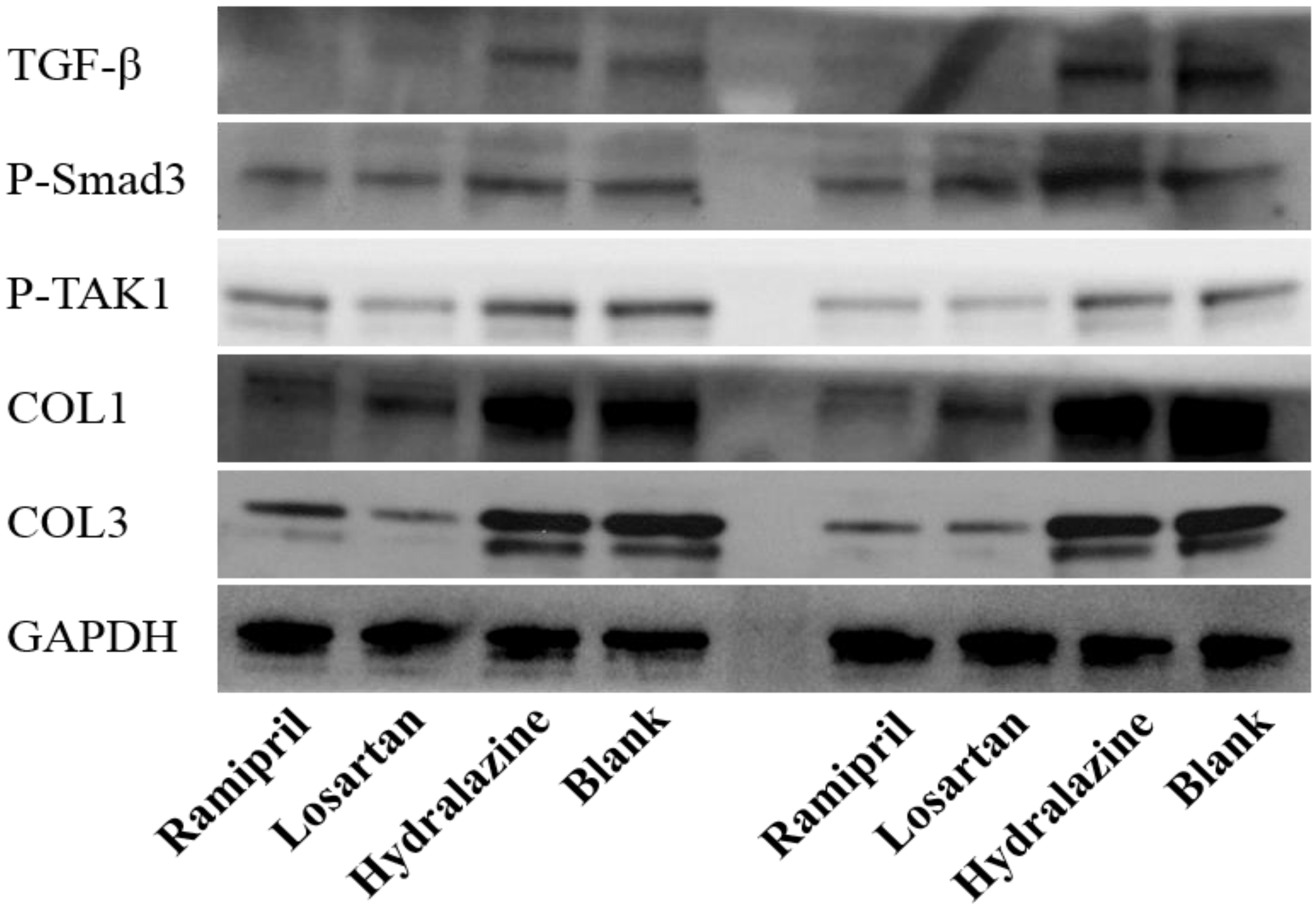
TGF-β
P-Smad3
P-TAK1
COL1
COL3
GAPDH
Ramipril
Losartan
Hydralazine
Blank
Ramipril
Losartan
Hydralazine
Blank

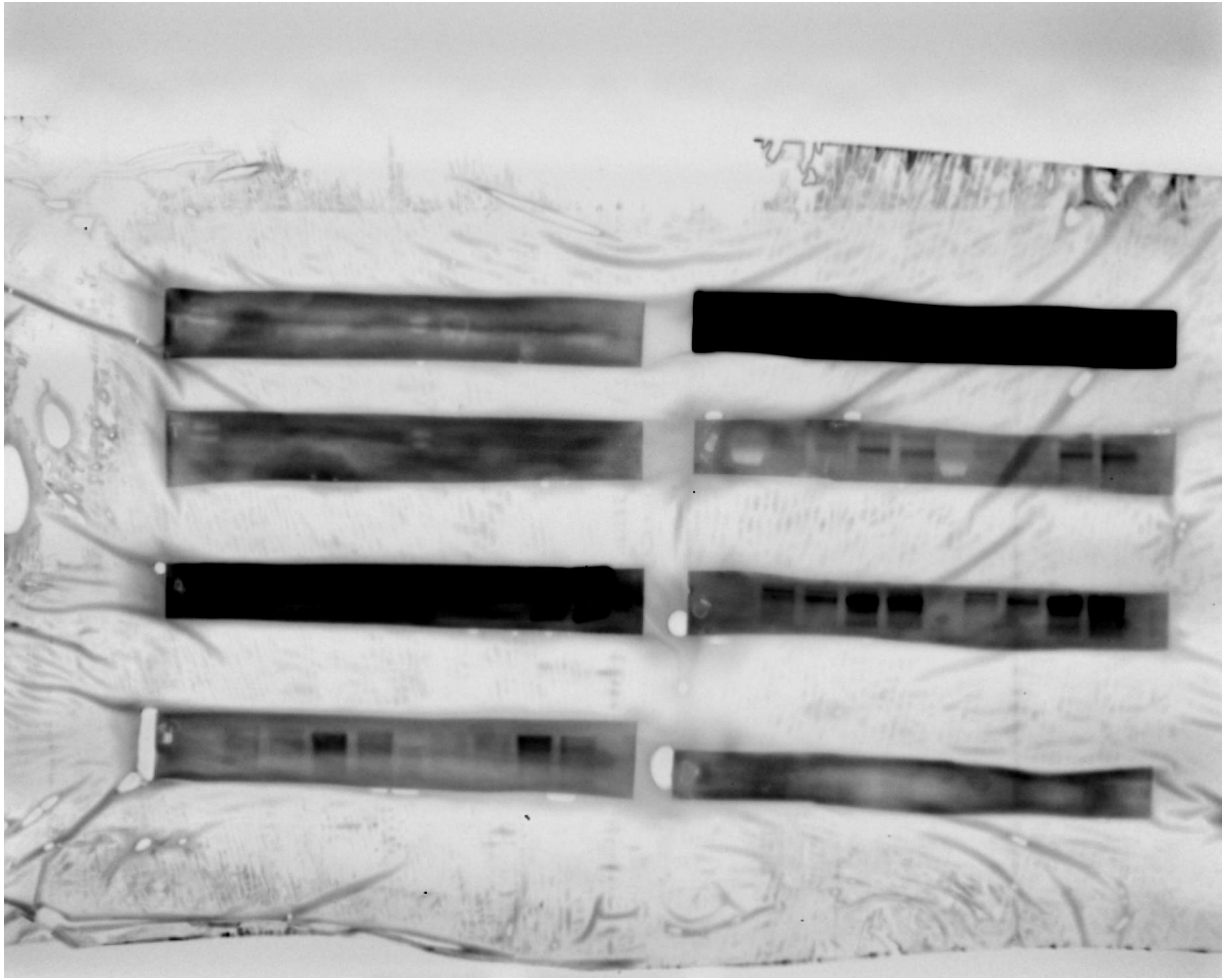

TGF-β

p-Smad2/3

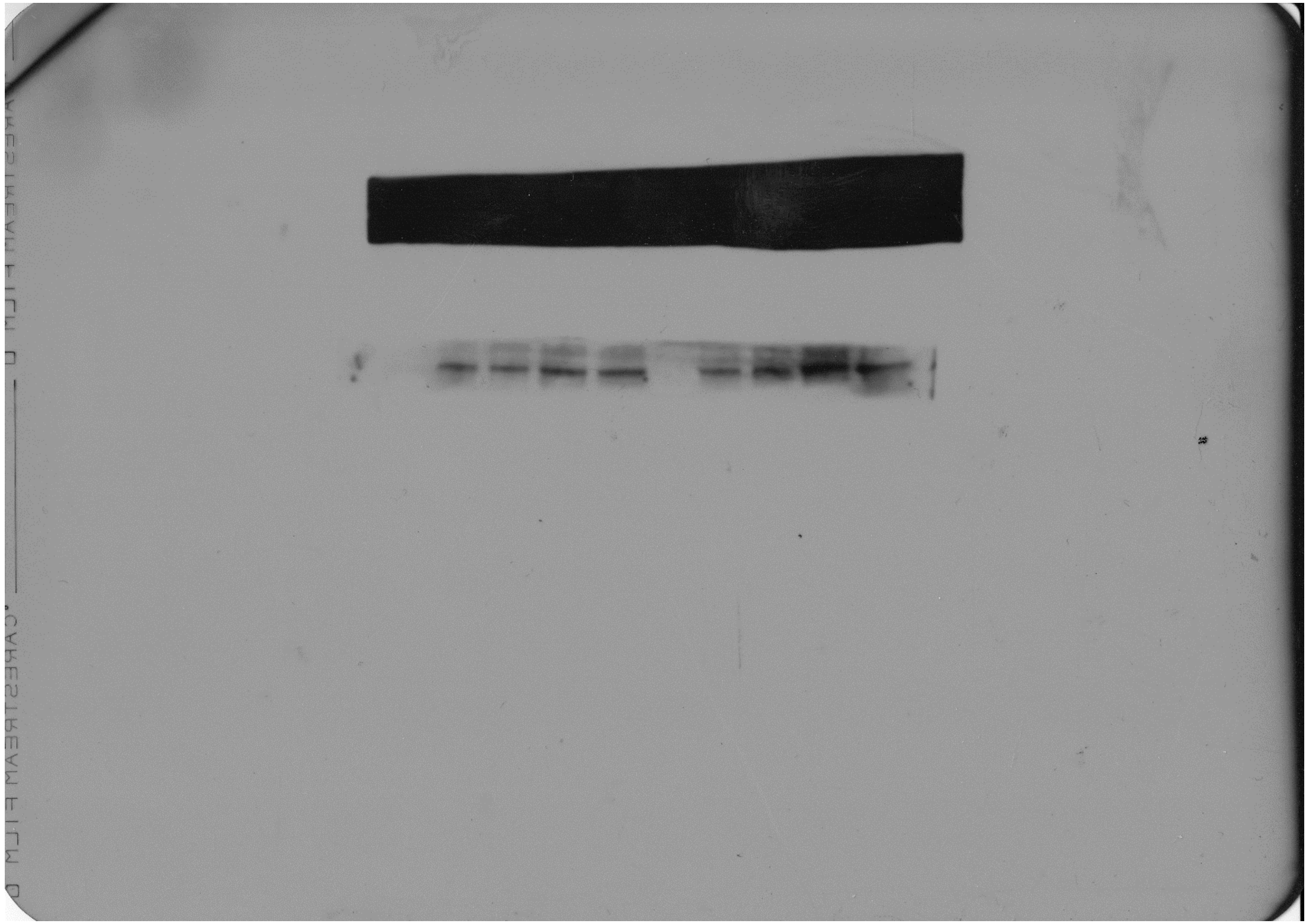

p-TAK1

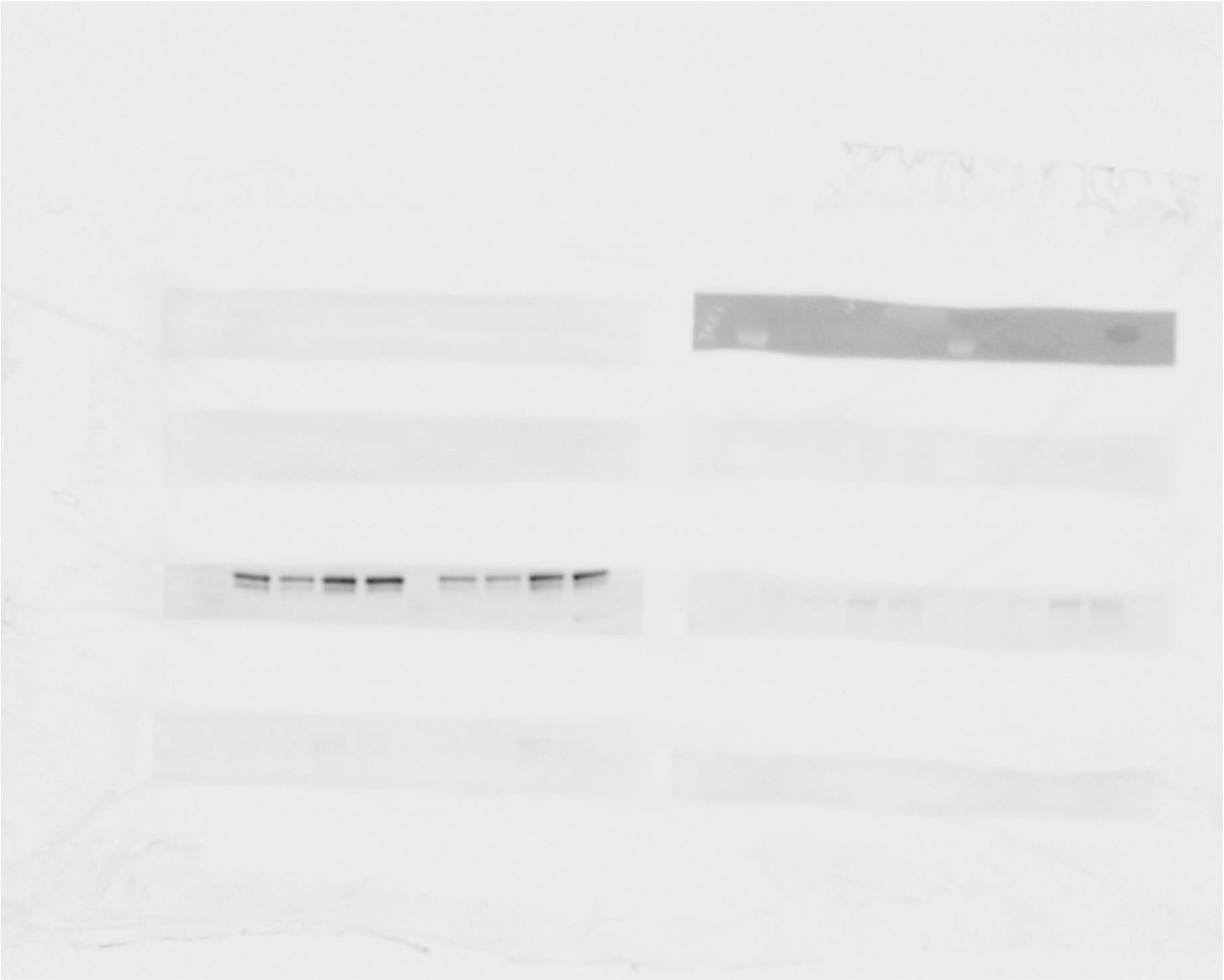

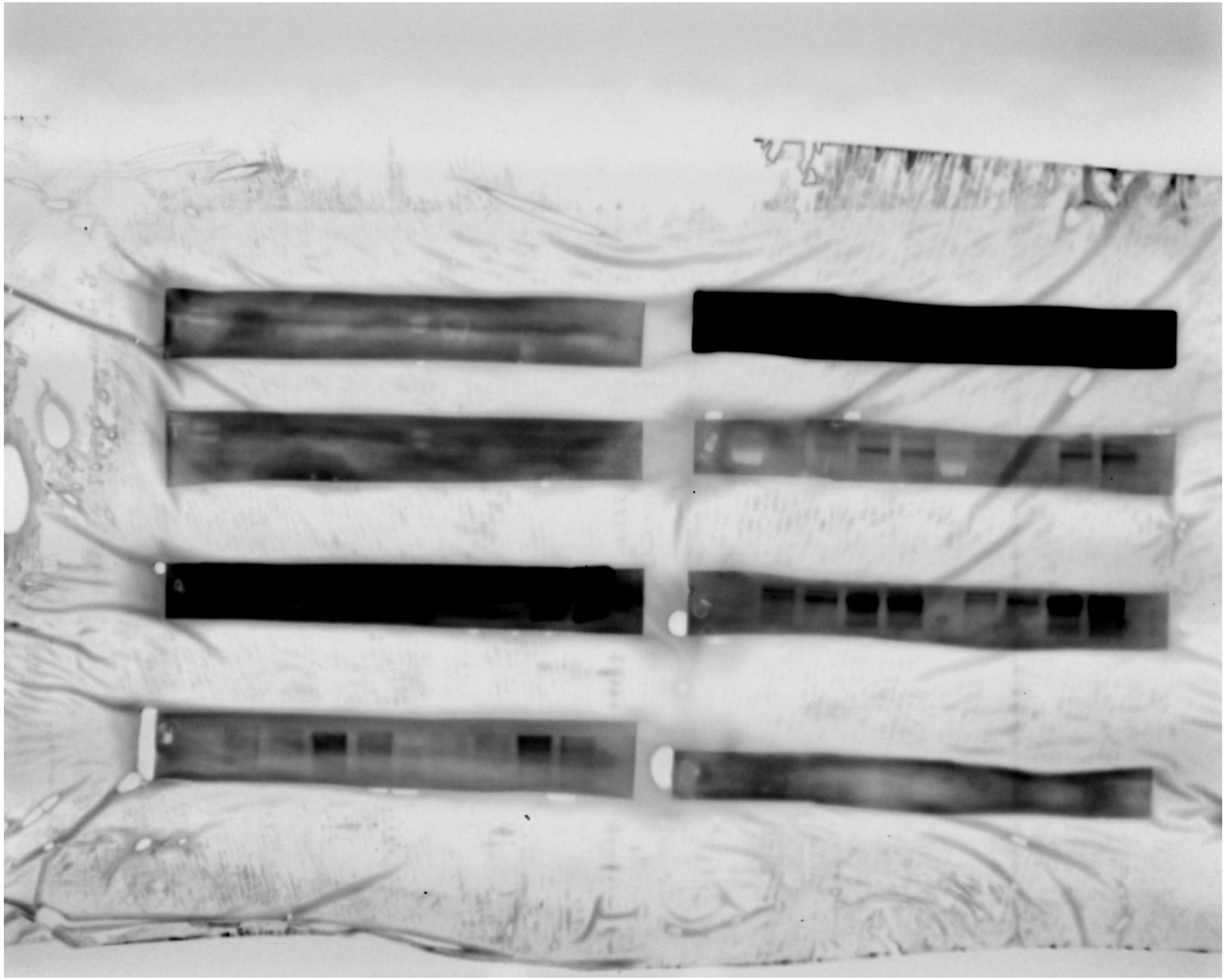

COL 1

COL 3

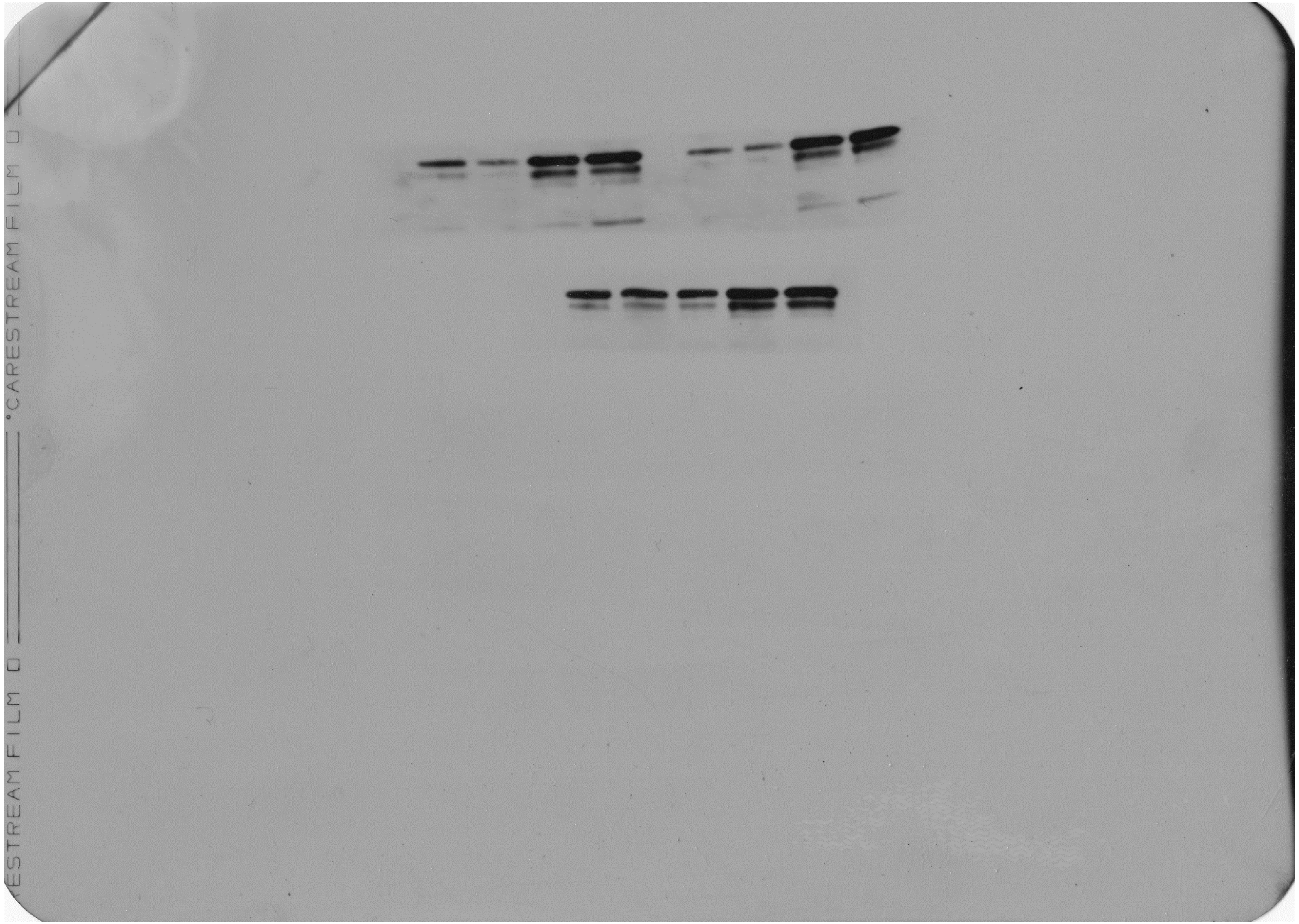

GAPDH

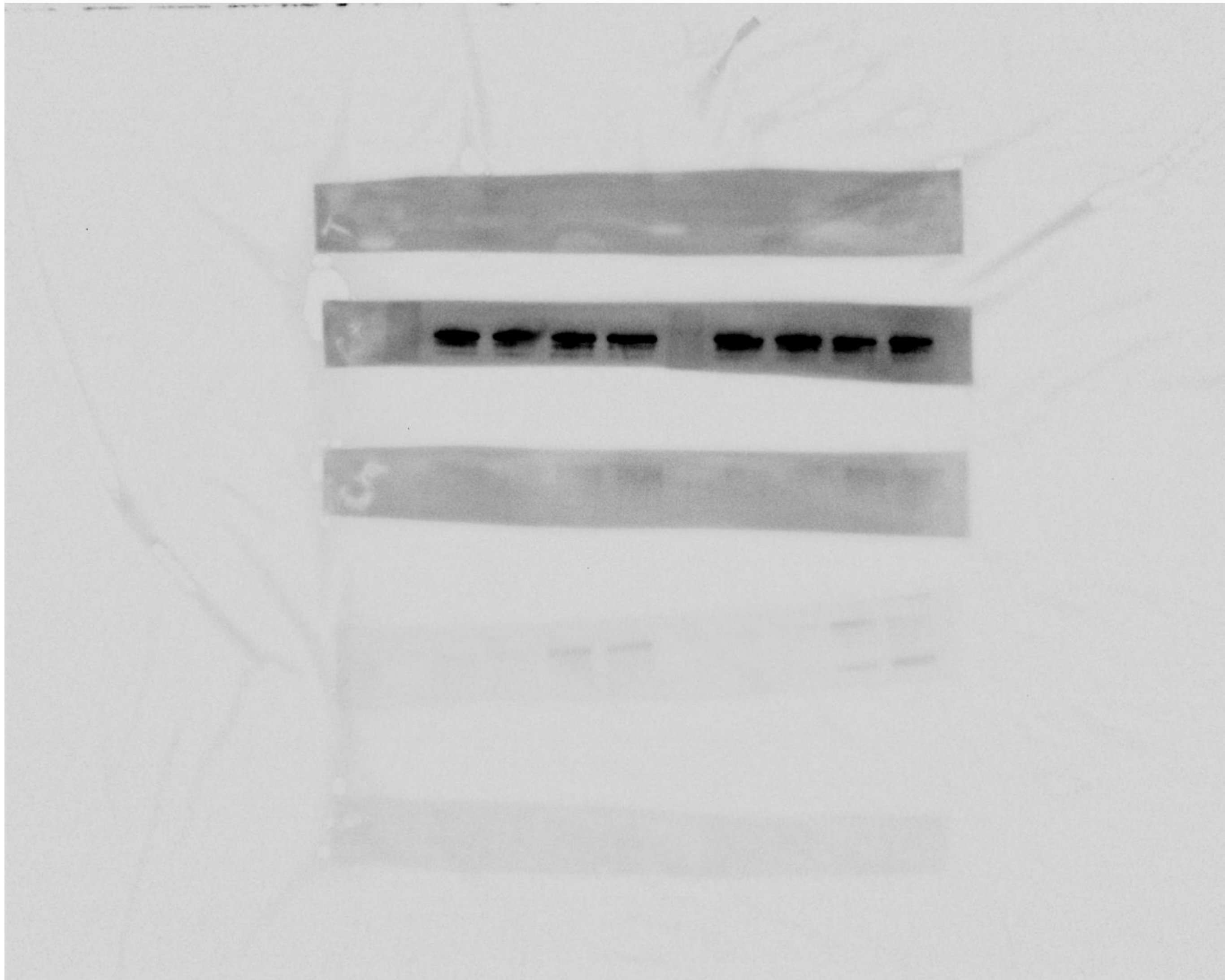

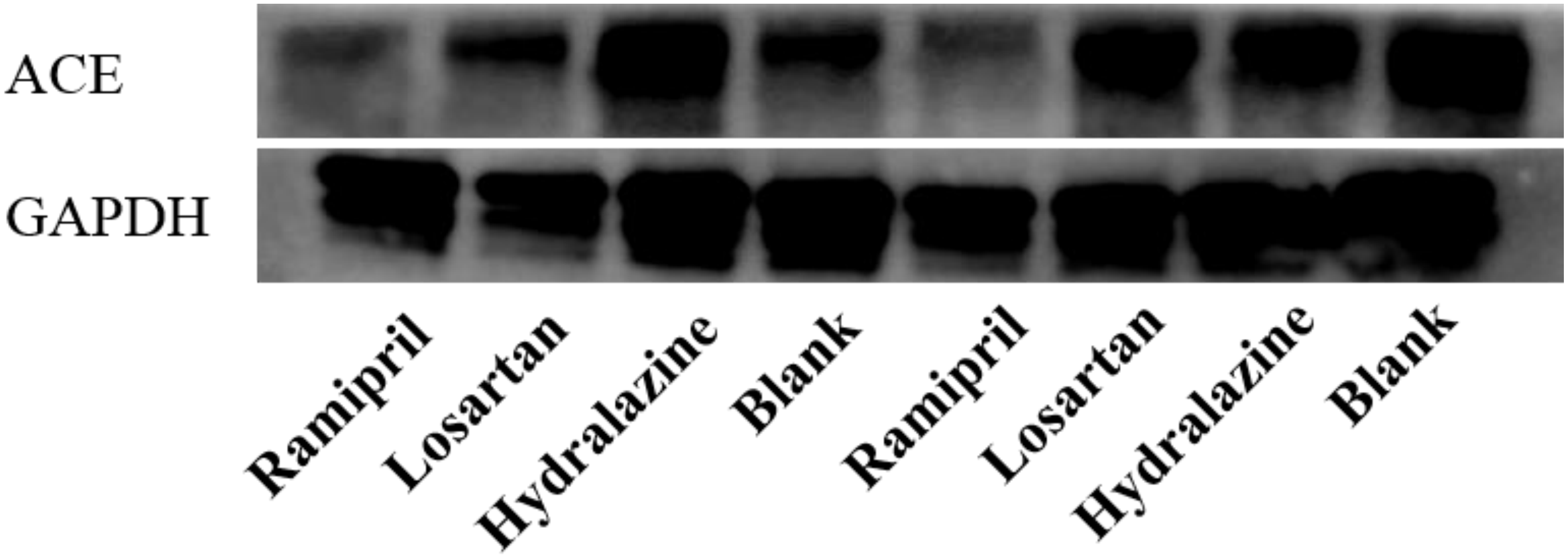
ACE
GAPDH
Ramipril
Losartan
Hydralazine
Blank
Ramipril
Losartan
Hydralazine
Blank

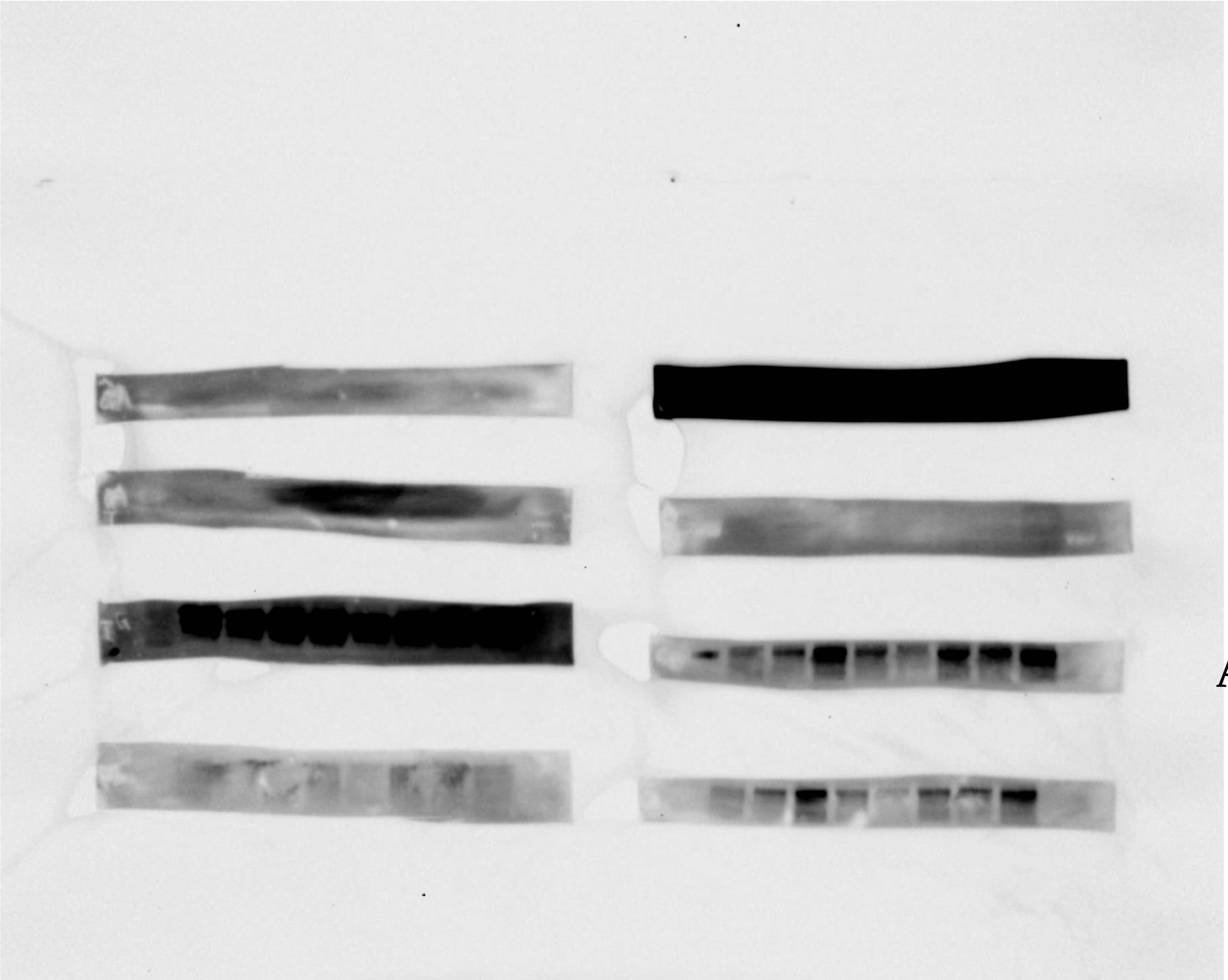

ACE

GAPDH

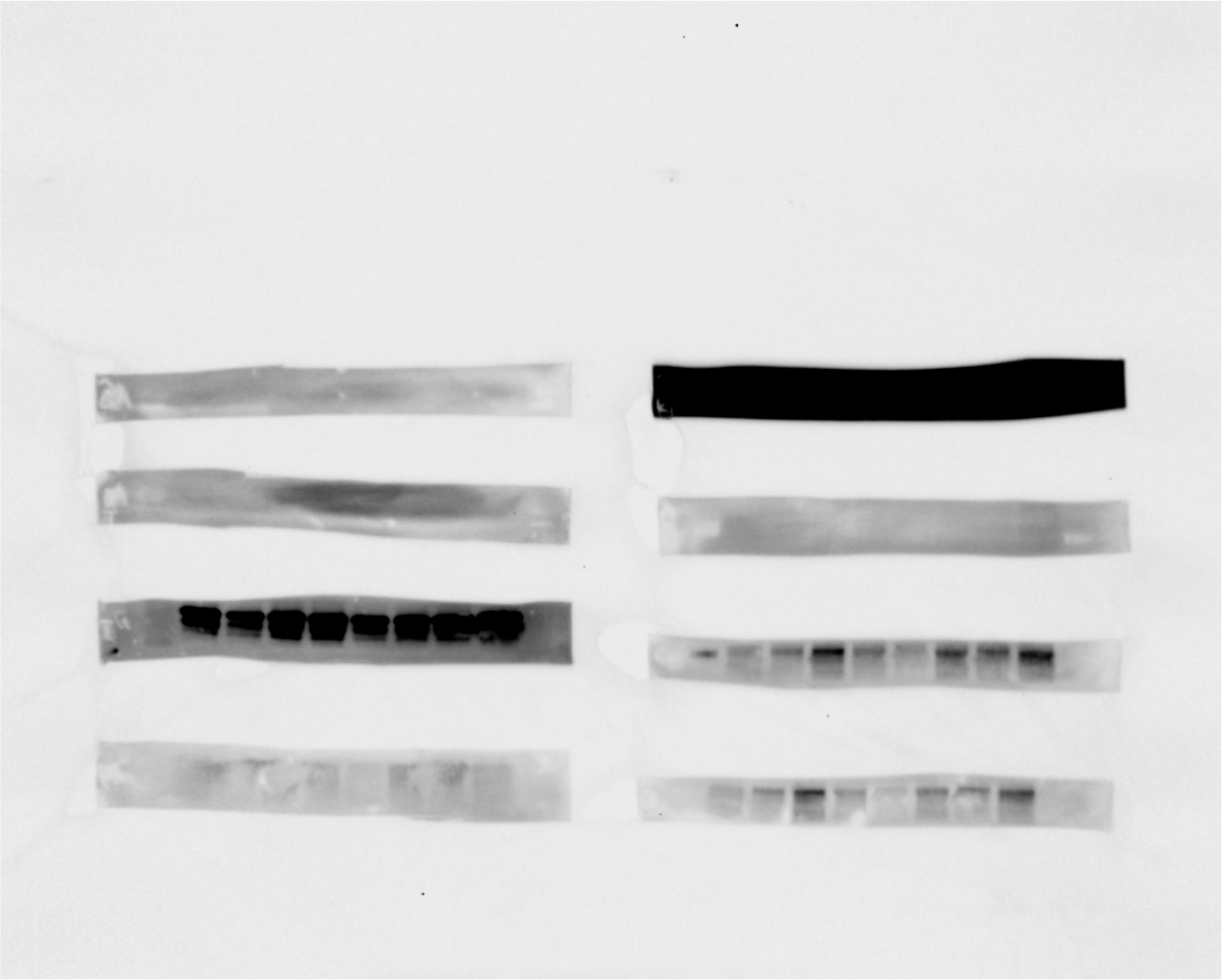

Supplement: Supplementary file 1 — supplementary figures [file 41598_2018_21600_MOESM1_ESM.pdf]
